# A rare case of anti-DPPX encephalitis combined with neuroleptospirosis

**DOI:** 10.1186/s12883-024-03538-x

**Published:** 2024-01-19

**Authors:** Yong Jin, Wei Lan, Xiaodong Chen, Wu Liu, Weiliang Luo, Suqin Chen

**Affiliations:** Department of Neurology, Huizhou Central People’s Hospital, No. 41, Eling North Road, Huizhou, Guangdong Province 516001 China

**Keywords:** Neuroleptospirosis, anti-DPPX encephalitis, Metagenomic next-generation sequencing (mNGS), Autoimmune encephalitis, Case report

## Abstract

**Background:**

Neuroleptospirosis and anti-dipeptidyl-peptidase-like protein 6 (DPPX) encephalitis are both very rare and have only been reported in the form of respective case reports. There are no reports of anti-DPPX encephalitis combined with neuroleptospirosis in the literature. We reported the first case of neuroleptospirosis combined with elevated DPPX antibodies in serum and cerebrospinal fluid (CSF).

**Case presentation:**

A previously healthy 53-year-old Chinese male farmer with a history of drinking raw stream water and flood sewage exposure was brought to the hospital due to an acute onset of neuropsychiatric symptoms. No fever or meningeal irritation signs were detected on physical examination. Routine laboratory investigations, including infection indicators, leukocyte and protein in CSF, electroencephalogram and gadolinium-enhanced magnetic resonance imaging of the brain, all revealed normal. While metagenomic next-generation sequencing (mNGS) identified the DNA genome of *Leptospira interrogans* in the CSF. Anti-DPPX antibody was detected both in blood and in CSF. A diagnosis of neuroleptospirosis combined with autoimmune encephalitis associated with DPPX-Ab was eventually made. He resolved completely after adequate amount of penicillin combined with immunotherapy.

**Conclusion:**

We highlight that in patients with acute or subacute behavioral changes, even in the absence of fever, if the most recent freshwater exposure is clear, physicians should pay attention to leptospirosis. Due to the low sensitivity of routine microscopy, culture, polymerase chain reaction and antibody testing, mNGS may have more advantages in diagnosing neuroleptospirosis. As autoimmune encephalitis can be triggered by various infections, neuroleptospirosis may be one of the causes of autoimmune encephalitis. Since neuronal antibody measurements themselves are not that common in neuroleptospirosis, future studies are needed to determine whether the detection of anti-DPPX antibodies is a rare event in leptospirosis. Early identification of autoimmune encephalitis and timely administration of immunotherapy may lead to a better outcome.

**Supplementary Information:**

The online version contains supplementary material available at 10.1186/s12883-024-03538-x.

## Introduction

Leptospirosis is a potentially severe zoonosis caused by the spirochete bacterium *Leptospira interrogans.* It usually gets transmitted when the water or floods contaminated with the urine or the blood of infected animals comes in contact with human skin or mucous membranes. Leptospirosis is characterised by a broad spectrum of clinical manifestations ranging from asymptomatic infection to fulminant and fatal disease. After an incubation period of 10 days (2–30 days) [1], patients may experience a phase with clinical spectrum ranges from the mild anicteric leptospirosis manifesting as an influenza-like presentation of fever and myalgia to the far more serious Weil’s syndrome, comprising jaundice, renal dysfunction, and bleeding diathesis [2]. Though it is very rare, leptospirosis may present as a primary neurological disease which only involves the nervous system, such as meningoencephalitis or aseptic meningitis, also called neuroleptospirosis [3].

Anti-dipeptidyl-peptidase-like protein 6 (DPPX) encephalitis, which was first described by Boronat et al. in 2013 [4], is a rare type of autoimmune encephalitis associated with weight loss, gastrointestinal symptoms, and neurological symptoms [5]. To date, only 65 patients with encephalitis associated with DPPX antibody have been described in the literature [6].

The mechanism of neurological manifestations caused by leptospirosis is still unclear. It is speculated that it may be essentially immune mediated [3]. There are a few reports of autoimmune encephalitis triggered by leptospira infection [7]. To the best of our knowledge, there are no reports of anti-DPPX encephalitis combined with neuroleptospirosis in the literature. Here, we report the first case of neuroleptospirosis complicated with DPPX antibody-associated encephalitis.

## Case presentation

A previously healthy 53-year-old Chinese male farmer was admitted to the hospital due to his “abnormal behavior for one day” on August 28, 2019, with the presentation of repetitive behavior, confusion, and irritability. He had a history of drinking raw stream water, flood sewage exposure and transient fever 1 week before admission. He was healthy before this episode and no medication history or drug abuse could be traced. There was no history of chronic diarrhea. Physical examination on admission revealed normal vital signs with a temperature of 36.8 ℃, respiratory rate of 20/minute, pulse of 74/minute, and blood pressure of 128/85 mmHg. He was in a sedative condition due to the application of sedative drugs in an external hospital. After the patient regained consciousness from sedatives, he presented with speech disorder, confusion and irritability. The Brudzinski sign and Kernig sign were negative.

Routine workup of blood to rule out other differential diagnosis, including metabolic disorder or endocrine disorder, were normal (see Supplementary Table 5, Additional File 1). Investigations for infection indicators, such as procalcitonin, serum human immunodeficiency virus antibody, syphilis rapid plasma reagin and herpes simplex virus antibody, were all normal. Tests for autoimmune antibodies and tumor markers of serum were negative (see Supplementary Table 4, Additional File 1). Gadolinium-enhanced magnetic resonance imaging of the brain was normal. No abnormalities were found in electromyography and electroencephalogram. Lumbar puncture showed that the cerebrospinal fluid (CSF) pressure was 120 mmH2O with normal leukocyte and protein (see Supplementary Table 3, Additional File 1).

**Fig. 1:**
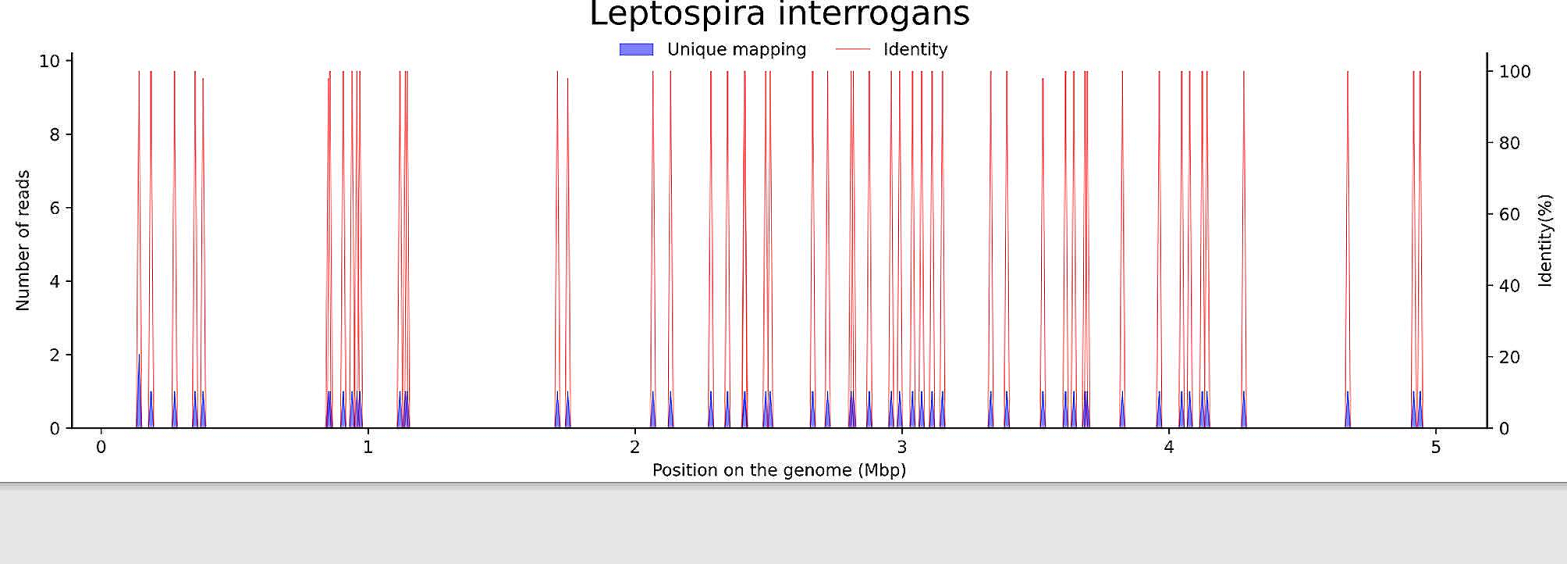
Sequence reads of *Leptospira interrogans* derived from the patient’s cerebrospinal fluid (CSF) specimen and the *Leptospira* genome in the reference database. The coverage map shown above is a mapping for a specific microorganism, reflecting the distribution of the sequence of the microorganism on its genome; the abscissa represents the size of the microbe’s gene group; and the ordinate represents the number of sequences detected in different genomic segments

In order to identify whether the patient had central nervous system (CNS) infection or autoimmune encephalitis, the patient’s CSF and peripheral blood samples were sent to the laboratories for metagenomic next-generation sequencing (mNGS) (BGI Gene Technology Company, Shenzhen, China) and autoimmune encephalitis related antibodies testing (Kindstar Global Medical Technology Co., Ltd, Beijing, China), respectively. mNGS revealed 53 sequencing reads of *Leptospira interrogans* in his CSF (Fig. 1) (method regarding mNGS was shown in Additional File 1 and Supplementary Table 1 and Table 2). Penicillin with a dose of 4.8 million units per 6 h was immediately initiated upon receiving mNGS report on September 1st, 2019. Olanzapine and risperidone were successively prescribed to relieve his psychiatric symptoms. But no improvement was observed in the following four day’s therapy. A real-time quantitative polymerase chain reaction (qPCR) assay targeting lipL32 (T033, Xi’an Tianlong Technology Co., Ltd, Xian, China) for detection of pathogenic *Leptospira spp* and specific *Leptospira* IgM and IgG antibodies test (ELISA kits, ESR125M, ESR125G, Virion-Serion, Germany) on peripheral blood were performed on September 5 (on the 9th day of admission, before prescribing of methylprednisolone), but they both revealed negative (methods were shown in Additional File 1).

**Fig. 2:**
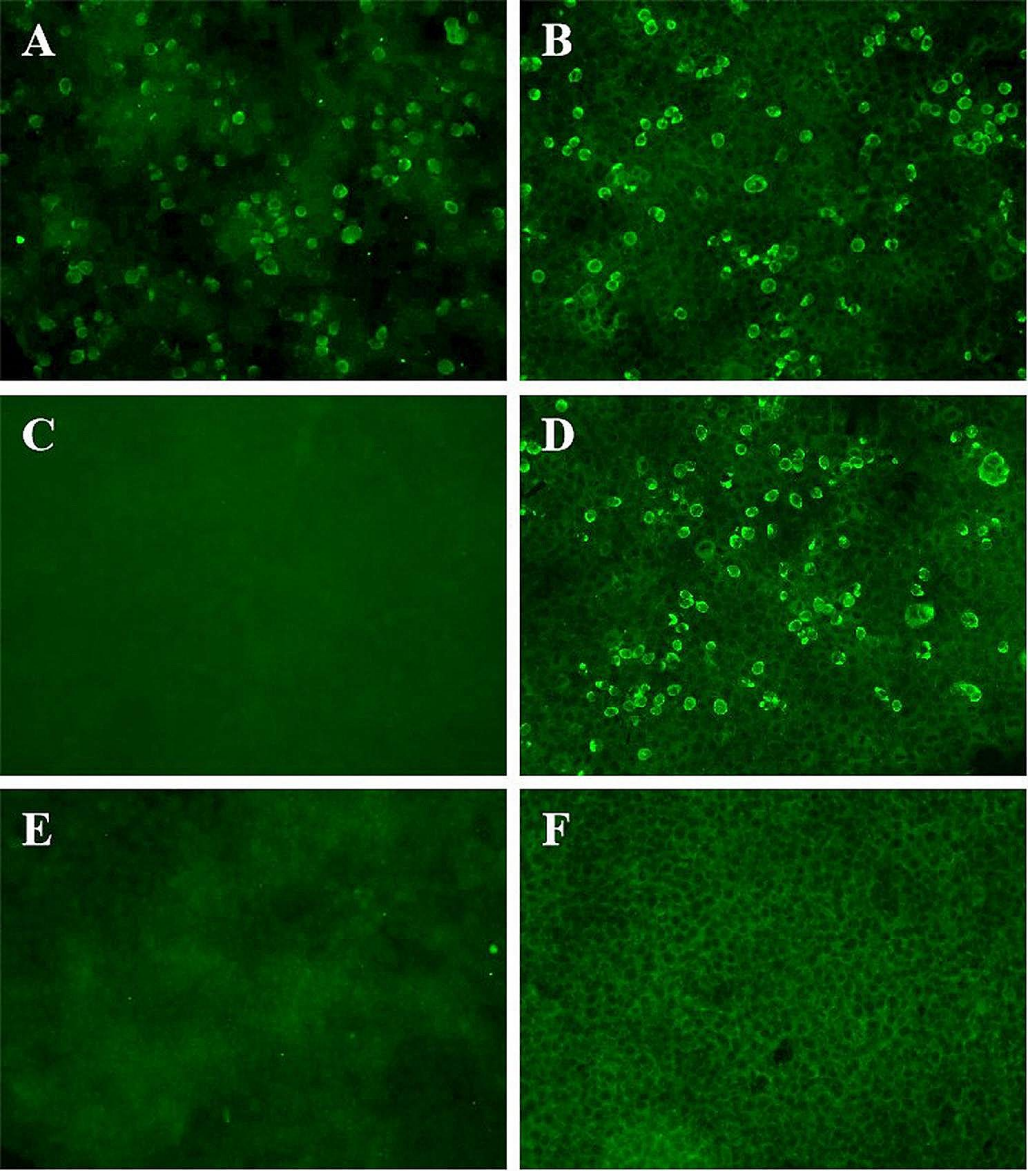
DPPX antibodies as detected by a cell-based fluorescence assay.Positive reaction with transfected HEK293 cells expressing DPPX after incubation with the patient’s CSF **(A)** (titer 1:1) and patient’s serum **(B)** (titer 1:32) before immunotherapy. Figures **C** and **D** were synchronous negative and positive controls, respectively. No DPPX antibodies were detected in the patient’s CSF **(E)** and blood **(F)** 20 days after immunotherapy

A DPPX IgG antibody detection kit (MT193-16, MYBiotech, Shanxi, China) with cell-based assay (CBA) was applied to capture IgG autoantibodies (protocols were shown in Additional File 1). And a report of positive IgG against DPPX both in blood (1:32) and CSF (1:1) (Fig. 2A and B) was received two days after the result of mNGS. Tests for other antibodies associated with tumors or autoimmune inflammatory disorders were all negative (see Supplementary Table 6, Additional File 1). The patient was eventually diagnosed with neuroleptospirosis combined with autoimmune encephalitis associated with DPPX-Ab. In addition to continuing with high-dose penicillin for 2 weeks, high-dose intravenous corticosteroids (methylprednisolone 1 g) for 5 days with subsequent slow dose tapering of prednisolone and immunoglobulin (0.4 g/kg per day) for a period of 5 days were also initiated on September 5, 2019. His mental symptoms relieved 2 days after the initiation of immunotherapy. After 20 days of treatment, his neurologic symptoms had substantially improved, and IgG against DPPX turned negative both in serum and CSF (Fig. 2C and D). The neuropsychiatric features completely resolved within 1 month after discharge. During the following 4-year follow-up, the patient had neither residual neurological symptoms nor recurrence.

## Discussion

Though neurologic manifestations of acute leptospirosis have been well documented and meningitis may be quite common in severe disease patients at late clinical course [2], rare patients present initially with primary neurological symptoms and lack of involvement in other systems [3, 8, 9]. For patients with endemic exposure present symptoms of anicteric or icteric leptospirosis, such as chills, headache, myalgia, abdominal pain, conjunctival suffusion, jaundice and acute renal failure, accurate laboratory tests, including cultures, serologic tests, and pathogen-specific PCR assays can provide confirmation in diagnosis. On the contrary, diagnosis of neuroleptospirosis can be challenging if it is not suspected in the clinical picture due to lacking of classical symptoms, such as the present patient. It is well known that the commonest manifestation of neuroleptospirosis is aseptic meningitis, which is responsible for 5–13% of all cases of aseptic meningitis [3, 10], typically characterised with headache, meningeal irritation and vomiting, along with elevated opening pressure, lymphocytic pleocytosis and raised protein in CSF. However, the case patient was characterized by encephalitis without meningeal irritation or abnormalities in CSF. Though he was suspected to be CNS infection, using conventional methods to establish a diagnosis could be very difficult since more than 100 different infectious agents can cause encephalitis [11, 12]. mNGS quickly provided a clinically actionable diagnosis of a specific pathogen, resulting in a favorable outcome in this case.

Leptospires are typically not detected by means of routine microscopy and culture of *Leptospira* is technically difficult with low sensitivity and long incubation period [2]. The standard diagnostic assay for leptospirosis detects the host’s serologic response [2, 13]. Though microscopic agglutination test (MAT) is the reference serological test for leptospirosis, it is usually positive in the later stages of the disease with a sensitivity of 44% in the first week and 82% in the second to fourth week of illness [14], which restricts its use for early diagnosis. In this case, we used a more sensitive IgM ELISA [13] as an alternative to detect the antibodies on the 9th day of disease onset before immunotherapy (the actual disease course may be longer since there was a transient fever one week before his admission), but the result was negative. Although it was probable that there were no antibodies in the blood since no involvement of other systems could be detected in addition to CNS, the antibody testing during convalescence should have been performed to confirm this hypothesis.

PCR as a more sensitive molecular diagnostic method allows detection of leptospires at early stage even in culture negative patient who has received an effective antimicrobial drug but have not cleared nonviable organism [15]. Conventional PCR assays have been replaced by qPCR which combines amplification and detection of amplified product in the same reaction vessel with excellent sensitivity and specificity and low contamination risk [14]. The qPCR test on blood in the case patient was negative. And it is possible that Leptospira DNA could be detected only in CSF and not in blood [16, 17]. Unfortunately, not enough CSF was available for qPCR testing in this case. mNGS, also known as high-throughput gene testing, offers numerous advantages in facilitating the diagnosis of suspected infected patients due to its unbiased sampling, which enables broad identification of known as well as unexpected pathogens or even the discovery of new organisms [18]. In this case, mNGS identified no other pathogens besides *L. interrogans* in the CSF, and no pathogens were detected in blood samples. Though the number of *L. interrogenes* genome reads (only 53 reads) were much smaller than previous report [17], it was the only pathogen specifically detected in the same batch of samples. Ruling out the possibility of contamination and given the patient’s history of sewage exposure, the diagnosis of neuroleptospirosis was considered. It seems necessary to use PCR to verify the diagnosis of mNGS. But in this case, a negative result is likely to be obtained since multiple negative PCR results were obtained in a previous reported case who had a much higher genome reads than the case patient [17].

The pathogenesis of leptospirosis has not been fully explained to date. It is believed that most of the clinical features are due to capillary endothelial damage and vasculitis. Although nervous system involvement is essentially thought to be immune mediated, few reports of autoimmune encephalitis triggered by leptospirosis could be traced in the published literature, in addition to four cases of acute disseminated encephalomyelitis (ADEM) and one case of anti-N-methyl-D-aspartate receptor (NMDAR) encephalitis [7]. To our knowledge, this is the first case of neuroleptospirosis combined with anti-DPPX encephalitis.

DPPX, a subunit of the Kv4.2 potassium channel, is expressed in neuronal dendrites and cell bodies. It is widely distributed in the CNS and in the gastrointestinal tract [19]. The clinical characteristics of anti-DPPX encephalitis are not fully understood, as only a few dozen cases have been reported. The majority of cases had a chronic course, with chronic diarrhea and prodromal weight loss that several months preceded the development of neurologic symptoms [5]. The enriched expression of DPPX in the myenteric plexus may explain the frequent gastrointestinal problems. A wide variety of neurologic symptoms indicating multifocal involvement of the CNS, including cognitive dysfunction, personality disorders, psychosis, hyperexcitability, brainstem or cerebellar dysfunction, sleep disorders, and sensory symptoms, usually gradually progress for months (range 1–54 months) before reaching the peak of the disease [5]. However, the present case was completely different from previously reported cases. He had an acute onset of neurologic symptoms, and no history of diarrhea or weight loss could be traced prior to the development of neurologic symptoms. In addition, the titer of anti-DPPX antibody in this patient was far lower than that in previously reported cases [5, 6, 20, 21]. Since various viral infections may trigger autoimmune encephalitis or immune-mediated neurological deterioration [7, 22], we speculated that the anti-DPPX antibodies in the case patient may be related to the antigen exposure and immune response caused by leptospirosis. The synergistic effect of the two diseases may explain the acute onset of psychosis symptoms. Furthermore, the low anti-DPPX antibody titer, which may indicate an early stage of the disease, should not be sufficient to decrease the density of surface DPPX to cause symptoms of diarrhea [5].

Additionally, we need to note that although cases of anti-NMDA antibody production in leptospirosis have been reported, the extent to which neuronal antibody production occurs in leptospirosis has not been clearly known. There were cases of leptospirosis characterized by behavioral changes [23, 24], but autoimmune antibodies were not investigated in these cases. Since neuronal antibody measurements themselves are not that common in neuroleptospirosis, future studies are needed to determine whether the detection of anti-DPPX antibodies is a rare event in leptospirosis.

Immunotherapy, as the standard treatment for autoimmune encephalitis, can also achieve good responses in most anti-DPPX encephalitis patients [5, 19]. Corticosteroids are the most commonly used first-line therapy [5]. Intravenous immunoglobulin (IVIg) therapy and plasma exchange are also widely used as other first-line therapies either given alone or in combination with corticosteroids. B-cell-targeted agents such as rituximab as second-line therapy are often needed for patients who have no responses to first-line therapies or relapse during dose tapering [5]. The case patient obtained a satisfactory response with corticosteroids combined with IVIg. No relapse was observed 4 years after drug withdrawal. As the immune-mediated process is considered the most likely pathogenesis behind neuroleptospirosis, immunotherapy could also reduce the severity and duration of illness.

## Conclusion

It is a great challenge to diagnose neuroleptospirosis due to the difficulty in detecting the pathogen or antibodies. We highlight that for patient with acute or subacute behavioral changes, even in the absence of fever, if the most recent freshwater exposure is clear, physicians should pay attention to leptospirosis. mNGS has great advantages in diagnosing neuroleptospirosis. As autoimmune encephalitis can be triggered by various infections, neuroleptospirosis may be one of the causes of autoimmune encephalitis. Since neuronal antibody measurements themselves are not that common in neuroleptospirosis, future studies are needed to determine whether the detection of anti-DPPX antibodies is a rare event in leptospirosis. Early identification and timely administration of immunotherapy may lead to a better outcome.

### Electronic supplementary material

Below is the link to the electronic supplementary material.

Supplementary Material 1

Supplementary Material 2

## Data Availability

Anonymous information related to the article can be obtained through the corresponding author upon reasonable request.
