# Supplementary material for: A rare case of anti-DPPX encephalitis combined with neuroleptospirosis
Source: BMC Neurol. 2024 Jan 19;24:34. doi: 10.1186/s12883-024-03538-x (PMC10797929; doi:10.1186/s12883-024-03538-x)
Supplement: Supplementary file 1 — Supplementary Material 1 [file 12883_2024_3538_MOESM1_ESM.doc]

**Supplementary Methods and Results**

**Metagenomic next-generation sequencing (mNGS) of clinical samples**

**Sample Processing and DNA Extraction**

Volume of 3 mL of blood were drawn from the patient, placed in blood collection tube and stored at room temperature for 3-5 minutes before plasma separation and centrifuged at 4,000 rpm for 10 min at 4℃ within 8 h of collection. Plasma samples were transferred to new sterile tubes. DNA was extracted from 300 uL of plasma using the TIANamp Micro DNA Kit (DP316, TIANGEN BIOTECH, Beijing, China) following the manufacturer’s operational manual. The extracted DNA specimens were used for the construction of DNA libraries [1].

1.5-3 mL CSF sample from the patient was collected according to standard procedures. 1.5 mL microcentrifuge tube with 0.6mL sample and 250μL 0.5mm glass bead were attached to a horizontal platform on a vortex mixer and agitated vigorously at 2800-3200 rpm for 30 min. Then 7.2 μL lysozyme was added for wall-breaking reaction. 0.3 mL sample was separated into a new 1.5mL microcentrifuge tube and DNA was extracted using the TIANamp Micro DNA Kit (DP316, TIANGEN BIOTECH, Beijing, China) according to the manufacturer’s recommendation.

**Sample Processing and RNA Extraction**

3 mL of blood were collected from the patient, within 8 hours the blood collection tube was centrifuged at 4℃ and 2910 rcf for 10 minutes, then 300 μL of plasma was taken to extract RNA following the manufacturer’s operational manual, using TIANMicrobe magnetic beads method pathogenic microorganism DNA/RNA extraction kit (NG550-01, WOOSEN BIOTECHNOLOGY, Yunnan, China).

1.5-3 mL of CSF was collected from the patient according to the standard sample collection procedure. 300 μL of supernatant was taken to extract RNA following the manufacturer’s operational manual, using TIANMicrobe magnetic beads method pathogenic microorganism DNA/RNA extraction kit (NG550-01, WOOSEN BIOTECHNOLOGY, Yunnan, China).

**Reverse transcription and two-strand synthesis**

The nucleic acid was subjected to fragmentation reaction, one-strand synthesis and two-strand synthesis to form double-stranded DNA nucleic acid, then purified by magnetic beads, and the purified DNA was used for DNA library construction [1].

**Construction of DNA libraries and Sequencing**

Then, DNA libraries were constructed through DNA-fragmentation, end-repair, adapter-ligation and PCR amplification. Agilent 2100 was used for quality control of the DNA libraries. Quality qualified libraries were pooled, DNA Nanoball (DNB) was made and sequenced by BGISEQ-50 /MGISEQ-2000 platform. [2]

**Bioinformatic analysis**

High-quality sequencing data were generated by removing low-quality reads, followed by computational substraction of human host sequences mapped to the human reference genome (hg19) using Burrows-Wheeler Alignment [3]. The remaining data by removal of low-complexity reads were classified by simultaneously aligning to Pathogens metagenomics Database (PMDB), consisting of bacteria, fungi, viruses and parasites. The classification reference databases were downloaded from NCBI (ftp://ftp.ncbi.nlm.nih.gov/genomes/).

**Supplementary Table 1 Summary counts of total mNGS reads**

| total reads | human reads | microorganisms reads | internal reference sequence |
| --- | --- | --- | --- |
| 15807173 | 69.02% | 7373 | detected |

**Supplementary Table 2 Summary counts of microorganism reads identified by DNA sequencing and RNA sequencing**

|  | Genus | reads | species | reads |
| --- | --- | --- | --- | --- |
| bacterium | *Leptospira* | 83 | *Leptospira interrogans* | 53 |
| fungus |  | 0 |  | 0 |
| virus |  | 0 |  | 0 |
| parasite |  | 0 |  | 0 |
| Mycobacterium tuberculosis complex |  | 0 |  | 0 |
| Mycoplasma/  chlamydia/  Rickettsia |  | 0 |  | 0 |
| Suspected background microorganisms* | *Ralstonia* | 53 | *Ralstonia insidiosa* | 17 |
|  | *Staphylococcus* | 48 | *Staphylococcus epidermidis* | 9 |
|  |  |  | *Staphylococcus hominis* | 7 |
|  | *Moraxella* | 46 | *Moraxella osloensis* | 45 |
|  | *Acidovorax* | 27 |  |  |
|  | *Cutibacterium* | 16 | *Cutibacterium* acnes | 11 |
|  | *Micrococcus* | 9 | *Micrococcus luteus* | 5 |
|  |  |  | *Micrococcus lylae* | 2 |
|  | *Acidovorax* | 7 | - | - |
|  | *Staphylococcus* | 4 | - | - |
|  | *Bacillus* | 3 | - | - |

*pathogen reads corresponded to the negative control sample, after alignment to the background bacterial library of the laboratory they were considered as background bacteria.

**Real time fluorescence quantitative PCR of *Leptospira***

A magnetic bead nucleic acid extraction kit (T033, Xi'an Tianlong Technology Co., Ltd, Xian, China) was used to extract DNA from the whole blood according to its instructions. Real time fluorescence quantitative PCR kit (Beijing Applied Biological Technologies Co.,Ltd, Beijing, China) was used to detect the DNA of *Leptospira* in the sample. The total volume of reaction system was 25 µL: 18 µL of reaction solution, 2 µL of polymerase, and 5 µL of template. The reaction conditions were: 95℃ for 5 minutes; 95℃ for 10 seconds, 60℃ for 40 seconds, 40 cycles (7500 Real time PCR system, Applied Biosystems, USA).

**Detection of specific IgM and IgG antibodies to *Leptospira***

Specific IgM and IgG antibodies to *Leptospira* were detected by ELISA kits (Virion-Serion, Germany). Detailed experimental procedure was carried out according to manufacturer’s operational manual.

**Detecting antibodies related to autoimmune encephalitis with cell-based assay (CBA)**

DPPX IgG antibody detection kit (MT193-16, MYBiotech, Shanxi, China) and other antibody detection kits (MYBiotech, Shanxi, China) with CBA was used to capture IgG autoantibodies following the instructions of the kit. Briefly, after rewarming of reagents and slides at room temperature for 10 minutes, 120 μL of sample was added to each reaction zone and incubated at room temperature for 1 hour. Then, 120 μL of diluted FITC labeled anti-human IgG antibody was added to each reaction zone and incubated at room temperature in dark for 30 minutes after removing the sample and rinsing at first step. Excess secondary antibodies would be removed and the slides were observed and took photos under a fluorescence microscope. If the test result was positive, the serum sample would be diluted in a ratio of 1:100 and 1:1000, and the CSF sample in a ratio of 1:10 and 1:100, which would be retested to determine the antibody titer.

**Enzyme linked immunospot assay (dot-ELISA) to detect antibodies related to autoimmune encephalitis**

Dot-ELISA antibody detection kits (MYBiotech, Shanxi, China) were used in this case. The experiment was performed following the manufacturer’s operational manual.

**Supplementary Table 3 Results of routine testing of CSF**

|  | result | reference range |
| --- | --- | --- |
| colour | Transparent |  |
| clot | none |  |
| leukocyte | 3.0*10^6^ cells/L | 0-8*10^6^ cells/L |
| erythrocyte | 0/HP |  |
| L-lactate dehydrogenase | 27 U/L | 114-225 U/L |
| trace total protein | 427 mg/L | 140-450 mg/L |
| chlorine | 127 mmol/L | 120-130 mmol/L |
| glucose | 4.37 mmol/L | 2.5-4.4 mmol/L |
| adenosine deaminase | 1 U/L | 4-20 U/L |
| tryptophan test | negative | negative |

**Supplementary Table 4 Tests for autoimmune antibodies and tumor related antigens in serum**

| items | result | reference range |
| --- | --- | --- |
| Anticardiolipin antibody-IgA (ACA-IgA) | negative | negative |
| Anticardiolipin antibody-IgM (ACA-IgM) | negative | negative |
| Anticardiolipin antibody-IgG (ACA-IgG) | negative | negative |
| pANCA | negative | negative |
| cANCA | negative | negative |
| MPO-ANCA IgG | negative | negative |
| PR3-ANCA IgG | negative | negative |
| Anti-GBM | negative | negative |
| Anti-nRNF/Sm | negative | negative |
| Anti-SM | negative | negative |
| Anti-SSA | negative | negative |
| Anti-Ro-52 | negative | negative |
| Anti-SSB | negative | negative |
| Anti-SCL-70 | negative | negative |
| Anti-JO-1 | negative | negative |
| Anti-CENPB | negative | negative |
| Anti-dsDNA | negative | negative |
| Anti-nucleosome | negative | negative |
| Anti-histones | negative | negative |
| anti-ribosomal p protein | negative | negative |
| CA72-4 | 2.130 U/ml | 0.000-6.900 |
| CA125 | 5.9 U/ml | 0.0-35.0 |
| CA15-3 | 12.8 U/ml | 0.0-25.0 |
| CA19-9 | 8.1 U/ml | 0-27 |
| alpha-fetoprotein | 2.44μg/L | 0-7 |
| carcinoembryonic antigen | 2.33μg/L | <5.0 |
| prostate-specific antigen | 0.594μg/L | 0-3.1 |
| ferritin | 490.4μg/L ↑ | 30-400 |

↑: The result was higher than reference value.

**Supplementary Table 5 Examinations to exclude endocrine, metabolic and other diseases**

| items | result | reference range |
| --- | --- | --- |
| free triiodothyronine (FT3) | 3.34 pmol/L | 3.28-6.47 |
| free thyroxine (FT4) | 12.26 pmol/L | 7.50-21.10 |
| high sensitivity thyrotropin (H-TSH) | 0.71 uIU/ml | 0.34-5.60 |
| triiodothyronine (T3) | 0.87 nmol/L | 1.01-2.48 |
| Thyroxine (T4) | 112.66 nmol/L | 78.38-157.40 |
| Thyroglobulin (TG) | 0.22 ng/ml | 0.00-30.00 |
| Hemoglobin (Hb) | 127 g/L | 130-175 |
| white blood cell count | 6.9*10^9^/L | 3.5-9.5 |
| platelet | 283*10^9^/L | 125-350 |
| rapid erythrocyte sedimentation rate (ESR) | 9 mm/H | 0-28 |
| Antiserum of streptolysin O (ASO) | 131 IU/ml | 0-200 |
| rheumatoid factor (RF) | 11.3 IU/ml | <14 |
| C-reactive protein (CRP) | 0.46 mg/L | 0.00-8.20 |
| Complement 3(C3) | 1.24 g/L | 0.79-1.52 |
| Complement 4 (C4) | 0.28 g/L | 0.16-0.38 |
| IgG | 25.21 g/L | 7.00-16.00 |
| IgM | 1.48 g/L | 0.40-2.30 |
| IgA | 1.67 g/L | 0.70-4.00 |
| lactic acid | 1.77 mmol/L | 0.50-2.20 |
| creatine kinase (CK) | 341 U/L ↑ | 38-174 |
| creatine kinase-MB | 17.6 U/L | 0-25 |
| Glucose | 5.8 mmol/L | 3.9-6.1 |
| Natrium (Na+) | 144 mmol/L | 137-147 |
| Potassium (K+) | 3.51 mmol/L | 3.5-5.3 |
| CO2 | 21.3 mmol/L | 22-29 |
| creatinine | 59 μmol/L | 62-115 |
| osmotic pressure | 289.07 mOsm/kg |  |
| albumin | 41 g/L | 40-55 |
| total bilirubin | 22.6 μmol/L ↑ | 5.1-17.1 |
| glutamic-pyruvic transaminase (ALT) | 24.7 U/L | 9-50 |
| glutamic oxaloacetic transaminase (AST) | 25.9 U/L | 15-40 |
| blood ammonia | 46 μmol/L | 16-60 |

↑: The result was higher than reference value.

**Supplementary Table 6** **Autoimmune encephalitis related antibodies and paraneoplastic syndrome related antibodies**

| antibody description | sample type | result | reference range | assay |
| --- | --- | --- | --- | --- |
| Anti-NMDAR IgG | blood | negative | negative | CBA |
|  | CSF | negative |  |  |
| Anti-AMPAR1 IgG | blood | negative | negative | CBA |
|  | CSF | negative |  |  |
| Anti-AMPAR2 IgG | blood | negative | negative | CBA |
|  | CSF | negative |  |  |
| Anti-LG1 IgG | blood | negative | negative | CBA |
|  | CSF | negative |  |  |
| Anti-CASPR2 IgG | blood | negative | negative | CBA |
|  | CSF | negative |  |  |
| Anti-GABABR IgG | blood | negative | negative | CBA |
|  | CSF | negative |  |  |
| Anti-DPPX IgG | blood | 1:32 positive | negative | CBA |
|  | CSF | 1:1 positive |  |  |
| Anti-IgLON5 IgG | blood | negative | negative | CBA |
|  | CSF | negative |  |  |
| Anti-GlyRα1 IgG | blood | negative | negative | CBA |
|  | CSF | negative |  |  |
| Anti-GABAARα1 IgG | blood | negative | negative | CBA |
|  | CSF | negative |  |  |
| Anti-GABAARβ3 IgG | blood | negative | negative | CBA |
|  | CSF | negative |  |  |
| Anti-mGluR5 IgG | blood | negative | negative | CBA |
|  | CSF | negative |  |  |
| Anti-D2R IgG | blood | negative | negative | CBA |
|  | CSF | negative |  |  |
| Anti-Neurexin3α IgG | blood | negative | negative | CBA |
|  | CSF | negative |  |  |
| Anti-Hu IgG | blood | negative | negative | dot-ELISA |
|  | CSF | negative |  |  |
| Anti-Yo IgG | blood | negative | negative | dot-ELISA |
|  | CSF | negative |  |  |
| Anti-Ri IgG | blood | negative | negative | dot-ELISA |
|  | CSF | negative |  |  |
| Anti-CV2 IgG | blood | negative | negative | dot-ELISA |
|  | CSF | negative |  |  |
| Anti-Ma2 IgG | blood | negative | negative | dot-ELISA |
|  | CSF | negative |  |  |
| Anti-Amphiphysin IgG | blood | negative | negative | dot-ELISA |
|  | CSF | negative |  |  |
| Anti-Ma1 IgG | blood | negative | negative | dot-ELISA |
|  | CSF | negative |  |  |
| Anti-SOX1 IgG | blood | negative | negative | dot-ELISA |
|  | CSF | negative |  |  |
| Anti-Tr (DNER) IgG | blood | negative | negative | dot-ELISA |
|  | CSF | negative |  |  |
| Anti-Zic4 IgG | blood | negative | negative | dot-ELISA |
|  | CSF | negative |  |  |
| Anti-GAD65 IgG | blood | negative | negative | dot-ELISA |
|  | CSF | negative |  |  |
| Anti-PKCγ IgG | blood | negative | negative | dot-ELISA |
|  | CSF | negative |  |  |
| Anti-Recoverin IgG | blood | negative | negative | dot-ELISA |
|  | CSF | negative |  |  |
| Anti-Titin IgG | blood | negative | negative | dot-ELISA |
|  | CSF | negative |  |  |

**References**

[1]. Long Y. ,Zhang Y.X.,et al.,Diagnosis of Sepsis with Cell-free DNA by Next-Generation Sequencing Technology in ICU Patients. Archives of Medical Research 47 (2016) 365e371

[2]. Jeon, Y.J., et al., The feasibility study of non-invasive fetal trisomy 18 and 21 detection with semiconductor sequencing platform. PLoS One, 2014. 9(10): p. e110240.

[3]. Li, H. and R. Durbin, Fast and accurate short read alignment with Burrows-Wheeler transform. Bioinformatics, 2009. 25(14): p. 1754-60.
