# Supplementary material for: A rare case of anti-DPPX encephalitis combined with neuroleptospirosis
Source: BMC Neurol. 2024 Jan 19;24:34. doi: 10.1186/s12883-024-03538-x (PMC10797929; doi:10.1186/s12883-024-03538-x)
Supplement: Supplementary file 2 — Supplementary Material 2 [file 12883_2024_3538_MOESM2_ESM.docx]

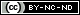

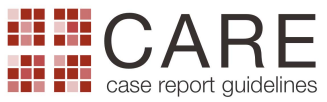
**CARE** **Checklist** **of** **information** **to** **include** **when** **writing** **a** **case** **report**

**Topic**

**Title**

**Key** **Words**

**Item** **Checklist** **item** **description** **Reported** **on** **Line**

**1** The diagnosis or intervention of primary focus followed by thewords“casereport” . . . . . . . . . . . . . . . . .. . . . . .. . . 1

**2** 2 to 5 key words that identify diagnoses or interventions in this case report, including "case report" . . . 63-64

**Abstract**

**(no** **references)**

**Introduction** **Patient** **Information**

**Clinical** **Findings** **Timeline**

**Diagnostic** **Assessment**

**Therapeutic** **Intervention**

**Follow-up** **and** **Outcomes**

**Discussion**

**Patient** **Perspective**

**3a** Introduction: What is unique about this case and what does it add to the scientific literature? . . . . . . . . . . . . . . . . 35-37

**3b** Mainsymptomsand/or important clinicalfindings .. . . . . . . . . . . . . . . . . . . . . . . . . . . . . . . . . . . . . . . . . . . . . . . . . . . .. . 41-46

47-49

**3c** Themaindiagnoses,therapeuticinterventions,andoutcomes............................ . . . . . . . . . . . . . . . **3d** Conclusion—Whatisthe main “take-away”lesson(s)from thiscase? . . . . . . . . . . . . . . . . . . . . . .. . . . . . . . . . . . . . .

67-89

51-61

1. One or two paragraphs summarizing why this case is unique (**may** **includereferences**) . . . . . . . . . . . . **5a** De-identified patient specific information. . . . . . . . . . . . . . . . . . . . . . . . . . . . . . . . . . . . . . . . . . .. . . . . . . . . **5b** Primary concerns and symptoms of the patient. . . . . . . . . . . .. . . . . . . . . . . . . . . . . . . . . . . . . . . . . . .. . . . . .

94-96

92-95

92

**5c** Medical, family, and psycho-social history including relevant genetic information . . . . . . . . . . . . . . . . . **5d** Relevant past interventions with outcomes . . . . . . . . . . . . . . . . . . . . .. . . . . . .. . . . . . . . . . . . . . . . . . . . . . .. .

97-103

99-101

**6** Describe significant physical examination (PE) and important clinical findings. . . . .. . . . . . . . . . . . . . . . . .

93,122,129,135

**7** Historical and current information from this episode of care organized as a timeline . . . . . . . . . . . . . . . **8a** Diagnostic testing (such as PE, laboratory testing, imaging, surveys). . . . . . . . . .. . . . . . . . . . . . .. . . . . . . .

114-117

104-113

**8b** Diagnostic challenges (such as access to testing, financial, or cultural) . . . . . . . . . . . . . . . . . .. . . . . . . . . . . **8c** Diagnosis (including other diagnoses considered) . . . . . . . . . . . . . . . . . . . . . . . . . . .. . . . . . . . . . . . . . . . . . . . . . **8d** Prognosis (such as staging in oncology) where applicable . . . . . . . . . . . . . . . . . . . . . . . .. . . . . . . . . . . . . . . . . **9a** Typesoftherapeuticintervention(suchaspharmacologic,surgical,preventive,self-care)................. . . . **9b** Administrationoftherapeuticintervention(suchasdosage,strength,duration) . . . . . . . . . . . . . . . . . . . . . . . . . . . . . **9c** Changesintherapeutic intervention(withrationale) . . . . . . . . . . . . . . . . . . . . . . . . . . . . . . . . . . . . . . . . . . . . . . . . . .. . **10a** Clinicianandpatient-assessedoutcomes(if available) . . . . . . .................. . . . . . . . . . . . . . .. . . . . . . . . . . . . **10b** Important follow-up diagnosticand othertest results . . . . . .. . . . . . . . . . . . . . . . . . . . . . . . . . . . . . . . . . . . . . . . . . .. . . **10c** Intervention adherence and tolerability (Howwasthisassessed?).. . . . . . . . . . . . . . . . . . . . . . . . . .. . . . . . . . . . . . . . **10d** Adverseandunanticipatedevents . . . . . . . . . . . . . . . . . . . . . . . . . . . . . . . . . . . . . . . . . . . . . . . . . . . . .. . . . . . . . . . . . . . **11a** A scientific discussionofthestrengths ANDlimitations associated withthis case report. . . . . . . . . . . . . . . . . . . . . . . **11b** Discussionoftherelevantmedicalliterature**with** **references**............................ . . . . . . . . . . . . . . **11c** Thescientific rationaleforany conclusions(includingassessmentofpossible causes) . . . . . .. . . . . . . . .. . . . . . . . . **11d** Theprimary“take-away”lessonsofthiscase report(without references) in a one paragraph conclusion. . . . . . .

140-142

104-109,114-115,138-139

NA

228-242

257-266

151-254

151-254

NA

140-142

145-146

143-148

121-123,140-142

140-142

143-148

**12** Thepatientshouldsharetheirperspectivein one to two paragraphs onthetreatment(s)theyreceived. . . . . . . .

**Informed** **Consent** **13** Didthepatientgiveinformedconsent?Pleaseprovideifrequested . . . . . . . . . . . . . . . . . . . . . . . . . . . . . . . . . . . . . . **Yes** **No**

√
